# Supplementary material for: Theoretical framework for mixed-potential-driven catalysis
Source: Commun Chem. 2024 Apr 1;7:69. doi: 10.1038/s42004-024-01145-y (PMC10985109; doi:10.1038/s42004-024-01145-y)
Supplement: Supplementary file 2 — Supplementary Information [file 42004_2024_1145_MOESM2_ESM.pdf]

Supplementary Information for

Theoretical framework for mixed-potential-driven catalysis

Mo Yan[1], Nuning Anugrah Putri Namari[1], Junji Nakamura*[2,3,4], Kotaro Takeyasu*[2,3,5]

[1]Graduate School of Science and Technology, University of Tsukuba, 1-1-1 Tennodai, Tsukuba, Ibaraki 305-8573, Japan.
[2]Department of Materials Science, Faculty of Pure and Applied Sciences, University of Tsukuba; 1-1-1 Tennodai, Tsukuba, Ibaraki 305-8573, Japan.
[3]Tsukuba Research Centre for Energy and Materials Science, University of Tsukuba; 1-1-1 Tennodai, Tsukuba, Ibaraki 305-8573, Japan.
[4]International Institute for Carbon-Neutral Energy Research (I[2]CNER), Kyushu University; 744 Motooka, Nishi-ku, Fukuoka-shi, Fukuoka 819-0395, Japan.
[5]R&D Center for Zero CO_2 Emission with Functional Materials, University of Tsukuba; 1-1-1 Tennodai, Tsukuba, Ibaraki 305-8573, Japan.

Corresponding authors:
nakamura.junji.700@m.kyushu-u.ac.jp; takeyasu.kotaro.gt@u.tsukuba.ac.jp

The PDF file includes:

Supplementary Text
Figures S1 to S5
Tables S1
Supplementary References

**Supplementary Information**	**Page**
**Supplementary Note 1:** Derivation of the mixed potential, the current at mixed potential, and the overpotential under different approximations	3
**Supplementary Note 2:** Adjustment of the overpotential partitioning based on the ratio of the exchange currents	6
**Supplementary Note 3:** Derivation of the Eqs. (23) - (25) for direction of the current flow or electron transfer between components I and II	8
**Supplementary Note 4:** Estimation of error when using Tafel and linear approximations	12
**Supplementary Note 5:** Derivation of Eq. (30) for the energy conversion pathway in the mixed-potential-driven catalysis	12
**Supplementary Note 6:** Expansion for general system with external electrochemical work	14
**Supplementary Note 7:** Effects of mass transport	14
**Supplementary Figure 1.** Illustration of the polarization curves to understand how to enhance the net reaction rate by adjusting the overpotential partitioning	16
**Supplementary Figure 2.** Estimation of error when using Tafel and linear approximations	17
**Supplementary Figure 3.** G vs. ξ plot at constant pressure and temperature	18
**Supplementary Figure 4.** Schematic diagram of the polarization curves and reaction current when there is external electrochemical work in the mixed-potential-driven catalysis	19
**Supplementary Figure 5.** Schematic plots of polarization curves for two half-reactions illustrate the mass transfer effect	20
**Supplementary Table 1.** The set values of parameters for solving the numerical value of mixed potential	21
**Supplementary References**	22

Supplementary Note 1: Derivation of the mixed potential, the current at mixed potential, and the overpotential under different approximations

We can derive the expressions for the mixed potential, the current at the mixed potential, and the overpotentials ($\left|\eta_1^{\text{mix}}\right|$ and $\left|\eta_2^{\text{mix}}\right|$) under different approximations. Here, we consider three approximations: (a) the two half-reactions have the same transfer coefficient, (b) Tafel approximation (different transfer coefficients), and (c) linear approximation of Taylor expansion.

We assume that the equilibrium potential of half-reaction 2 is greater than that of half-reaction 1 ($\phi_2^{\text{eq}} > \phi_1^{\text{eq}}$). This means that half-reaction 1 proceeds in the anodic oxidation direction, while half-reaction 2 proceeds in the cathodic reduction direction.

(a) The two half-reactions have the same transfer coefficient ($\boldsymbol{\alpha_1 = \alpha_2 = \alpha}$)

In mixed-potential-driven catalytic reaction systems in which $\alpha_1 = \alpha_2 = \alpha$ exists, the condition that the algebraic sum of currents for half-reaction 1 and 2 on component I and II is zero becomes:

$$
\begin{aligned}
i_1^{\text{I}\,0}\left(e^{(1-\alpha)f\eta_1^{\text{mix}}} - e^{-\alpha f\eta_1^{\text{mix}}}\right) + i_1^{\text{II}\,0}\left(e^{(1-\alpha)f\eta_1^{\text{mix}}} - e^{-\alpha f\eta_1^{\text{mix}}}\right)& \\
+ i_2^{\text{I}\,0}\left(e^{(1-\alpha)f\eta_2^{\text{mix}}} - e^{-\alpha f\eta_2^{\text{mix}}}\right)& \\
+ i_2^{\text{II}\,0}\left(e^{(1-\alpha)f\eta_2^{\text{mix}}} - e^{-\alpha f\eta_2^{\text{mix}}}\right) = 0&
\end{aligned}
\qquad \text{(S1-1)}
$$

The overpotentials upon the formation of mixed potential, η_1^{mix} and η_2^{mix}, are defined as:

$$
\eta_1^{\text{mix}} = \phi^{\text{mix}} - \phi_1^{\text{eq}} \qquad \text{(S1-2)}
$$

$$
\eta_2^{\text{mix}} = \phi^{\text{mix}} - \phi_2^{\text{eq}} \qquad \text{(S1-3)}
$$

At the condition that $\phi_2^{\text{eq}} > \phi_1^{\text{eq}}$, η_1^{mix} and η_2^{mix} are positive and negative, resepectively. Substituting overpotentials into Eq. (S1-1) gives:

$$
\begin{aligned}
\left(i_1^{\text{I}\,0} + i_1^{\text{II}\,0}\right)\left(e^{(1-\alpha)f(\phi^{\text{mix}}-\phi_1^{\text{eq}})} - e^{-\alpha f(\phi^{\text{mix}}-\phi_1^{\text{eq}})}\right)& \\
+ \left(i_2^{\text{I}\,0} + i_2^{\text{II}\,0}\right)\left(e^{(1-\alpha)f(\phi^{\text{mix}}-\phi_2^{\text{eq}})} - e^{-\alpha f(\phi^{\text{mix}}-\phi_2^{\text{eq}})}\right) = 0&
\end{aligned}
\qquad \text{(S1-4)}
$$

By extracting the exponential terms of mixed potential $e^{(1-\alpha)f\phi^{\text{mix}}}$ and $e^{-\alpha f\phi^{\text{mix}}}$, one can rearrange Eq. (S1-4) to Eq. (S1-5):

$$
\begin{aligned}
e^{(1-\alpha)f\phi^{\text{mix}}}\left(i_1^{\text{I}\,0} + i_1^{\text{II}\,0}\right)e^{-(1-\alpha)f\phi_1^{\text{eq}}} - e^{-\alpha f\phi^{\text{mix}}}\left(i_1^{\text{I}\,0} + i_1^{\text{II}\,0}\right)e^{\alpha f\phi_1^{\text{eq}}}& \\
+ e^{(1-\alpha)f\phi^{\text{mix}}}\left(i_2^{\text{I}\,0} + i_2^{\text{II}\,0}\right)e^{-(1-\alpha)f\phi_2^{\text{eq}}}& \\
- e^{-\alpha f\phi^{\text{mix}}}\left(i_2^{\text{I}\,0} + i_2^{\text{II}\,0}\right)e^{\alpha f\phi_2^{\text{eq}}} = 0&
\end{aligned}
\qquad \text{(S1-5)}
$$

Then, by organizing $e^{(1-\alpha)f\phi^{\text{mix}}}$ terms and $e^{-\alpha f\phi^{\text{mix}}}$ terms to the left-hand and right-hand sides of the equation, respectively, Eq. (S1-5) can be reduced to:

$$
\begin{aligned}
e^{(1-\alpha)f\phi^{\text{mix}}}\left[\left(i_1^{\text{I}\,0} + i_1^{\text{II}\,0}\right)e^{-(1-\alpha)f\phi_1^{\text{eq}}} + \left(i_2^{\text{I}\,0} + i_2^{\text{II}\,0}\right)e^{-(1-\alpha)f\phi_2^{\text{eq}}}\right]& \\
= e^{-\alpha f\phi^{\text{mix}}}\left[\left(i_1^{\text{I}\,0} + i_1^{\text{II}\,0}\right)e^{\alpha f\phi_1^{\text{eq}}} + \left(i_2^{\text{I}\,0} + i_2^{\text{II}\,0}\right)e^{\alpha f\phi_2^{\text{eq}}}\right]&
\end{aligned}
\qquad \text{(S1-6)}
$$

Thus, the mixed potential ϕ^{mix} is given by:

$$
\phi^{\text{mix}} = \frac{1}{f}\ln\frac{\left(i_1^{\text{I}\,0} + i_1^{\text{II}\,0}\right)e^{\alpha f\phi_1^{\text{eq}}} + \left(i_2^{\text{I}\,0} + i_2^{\text{II}\,0}\right)e^{\alpha f\phi_2^{\text{eq}}}}{\left(i_1^{\text{I}\,0} + i_1^{\text{II}\,0}\right)e^{-(1-\alpha)f\phi_1^{\text{eq}}} + \left(i_2^{\text{I}\,0} + i_2^{\text{II}\,0}\right)e^{-(1-\alpha)f\phi_2^{\text{eq}}}} \qquad \text{(S1-7)}
$$

Eq. (S1-7) indicates that the mixed potential ϕ^{mix} must be located between ϕ_1^{eq} and ϕ_2^{eq}. Substituting the obtained ϕ^{mix} of Eq. (S1-7) into the current of reaction 1 or reaction 2 gives the current at mixed potential, i^{mix}. In this context, we present the expression for i^{mix} using the current of the anodic reaction 1, represented as $i_1^{\mathrm{I}} + i_1^{\mathrm{II}}$:

$$\begin{aligned} i^{\mathrm{mix}} &= \left|i_1^{\mathrm{I}} + i_1^{\mathrm{II}}\right| = \left(i_1^{\mathrm{I}\,0} + i_1^{\mathrm{II}\,0}\right)\left(e^{(1-\alpha)f\eta_1^{\mathrm{mix}}} - e^{-\alpha f\eta_1^{\mathrm{mix}}}\right) \\ &= \left(i_1^{\mathrm{I}\,0} + i_1^{\mathrm{II}\,0}\right)\left[\left(\frac{\left(i_1^{\mathrm{I}\,0} + i_1^{\mathrm{II}\,0}\right) + \left(i_2^{\mathrm{I}\,0} + i_2^{\mathrm{II}\,0}\right)e^{\alpha f(\phi_2^{\mathrm{eq}}-\phi_1^{\mathrm{eq}})}}{\left(i_1^{\mathrm{I}\,0} + i_1^{\mathrm{II}\,0}\right) + \left(i_2^{\mathrm{I}\,0} + i_2^{\mathrm{II}\,0}\right)e^{-(1-\alpha)f(\phi_2^{\mathrm{eq}}-\phi_1^{\mathrm{eq}})}}\right)^{1-\alpha} \right. \\ &\qquad \left. - \left(\frac{\left(i_1^{\mathrm{I}\,0} + i_1^{\mathrm{II}\,0}\right) + \left(i_2^{\mathrm{I}\,0} + i_2^{\mathrm{II}\,0}\right)e^{\alpha f(\phi_2^{\mathrm{eq}}-\phi_1^{\mathrm{eq}})}}{\left(i_1^{\mathrm{I}\,0} + i_1^{\mathrm{II}\,0}\right) + \left(i_2^{\mathrm{I}\,0} + i_2^{\mathrm{II}\,0}\right)e^{-(1-\alpha)f(\phi_2^{\mathrm{eq}}-\phi_1^{\mathrm{eq}})}}\right)^{-\alpha}\right] \end{aligned} \tag{S1-8}$$

The absolute value of overpotentials $\left|\eta_1^{\mathrm{mix}}\right|$ and $\left|\eta_2^{\mathrm{mix}}\right|$ can be written by using the ϕ^{mix} of Eq. (S1-7) as below:

$$\left|\eta_1^{\mathrm{mix}}\right| = \phi^{\mathrm{mix}} - \phi_1^{\mathrm{eq}} = \frac{1}{f}\ln\frac{e^{\alpha f(\phi_2^{\mathrm{eq}}-\phi_1^{\mathrm{eq}})} + \dfrac{\left(i_1^{\mathrm{I}\,0} + i_1^{\mathrm{II}\,0}\right)}{\left(i_2^{\mathrm{I}\,0} + i_2^{\mathrm{II}\,0}\right)}}{e^{-(1-\alpha)f(\phi_2^{\mathrm{eq}}-\phi_1^{\mathrm{eq}})} + \dfrac{\left(i_1^{\mathrm{I}\,0} + i_1^{\mathrm{II}\,0}\right)}{\left(i_2^{\mathrm{I}\,0} + i_2^{\mathrm{II}\,0}\right)}} \tag{S1-9}$$

$$\left|\eta_2^{\mathrm{mix}}\right| = \phi_2^{\mathrm{eq}} - \phi^{\mathrm{mix}} = \frac{1}{f}\ln\frac{e^{(1-\alpha)f(\phi_2^{\mathrm{eq}}-\phi_1^{\mathrm{eq}})} + \dfrac{\left(i_2^{\mathrm{I}\,0} + i_2^{\mathrm{II}\,0}\right)}{\left(i_1^{\mathrm{I}\,0} + i_1^{\mathrm{II}\,0}\right)}}{e^{-\alpha f(\phi_2^{\mathrm{eq}}-\phi_1^{\mathrm{eq}})} + \dfrac{\left(i_2^{\mathrm{I}\,0} + i_2^{\mathrm{II}\,0}\right)}{\left(i_1^{\mathrm{I}\,0} + i_1^{\mathrm{II}\,0}\right)}} \tag{S1-10}$$

Eqs. (S1-9) and (S1-10) indicate that the kinetic parameter, ratio of exchange current $\left(i_2^{\mathrm{I}\,0} + i_2^{\mathrm{II}\,0}\right):\left(i_1^{\mathrm{I}\,0} + i_1^{\mathrm{II}\,0}\right)$, determines the total overpotential partitioning in mixed-potential-driven catalysis.

(b) Tafel approximation (different transfer coefficients)

Now we consider the case that both overpotentials are large enough $(|\eta|)RT/F$), such that the reverse reaction current can be ignored. Under this condition, the requirement for the sum of the four currents to be zero is as follows:

$$i_1^{\mathrm{I}\,0}e^{(1-\alpha_1)f\eta_1^{\mathrm{mix}}} + i_1^{\mathrm{II}\,0}e^{(1-\alpha_1)f\eta_1^{\mathrm{mix}}} - i_2^{\mathrm{I}\,0}e^{-\alpha_2 f\eta_2^{\mathrm{mix}}} - i_2^{\mathrm{II}\,0}e^{-\alpha_2 f\eta_2^{\mathrm{mix}}} = 0 \tag{S1-11}$$

Again, substituting overpotentials into Eq. (S1-11) yields:

$$\left(i_1^{\mathrm{I}\,0} + i_1^{\mathrm{II}\,0}\right)e^{(1-\alpha_1)f(\phi^{\mathrm{mix}}-\phi_1^{\mathrm{eq}})} = \left(i_2^{\mathrm{I}\,0} + i_2^{\mathrm{II}\,0}\right)e^{-\alpha_2 f(\phi^{\mathrm{mix}}-\phi_2^{\mathrm{eq}})} \tag{S1-12}$$

which can be rearranged to

$$\begin{aligned} &e^{(1-\alpha_1)f\phi^{\mathrm{mix}}}\left(i_1^{\mathrm{I}\,0} + i_1^{\mathrm{II}\,0}\right)e^{-(1-\alpha_1)f\phi_1^{\mathrm{eq}}} \\ &\qquad = e^{-\alpha_2 f\phi^{\mathrm{mix}}}\left(i_2^{\mathrm{I}\,0} + i_2^{\mathrm{II}\,0}\right)e^{\alpha_2 f\phi_2^{\mathrm{eq}}} \end{aligned} \tag{S1-13}$$

Eq. (S1-13) can be rewritten as:

$$\frac{e^{(1-\alpha_1)f\phi^{\mathrm{mix}}}}{e^{-\alpha_2 f\phi^{\mathrm{mix}}}} = \frac{\left(i_2^{\mathrm{I}\,0} + i_2^{\mathrm{II}\,0}\right) e^{\alpha_2 f\phi_2^{\mathrm{eq}}}}{\left(i_1^{\mathrm{I}\,0} + i_1^{\mathrm{II}\,0}\right) e^{-(1-\alpha_1)f\phi_1^{\mathrm{eq}}}} \tag{S1-14}$$

Thus, the mixed potential with Tafel approximation is given by:

$$\phi^{\mathrm{mix}} = \frac{1}{(1-\alpha_1+\alpha_2)f} \ln \frac{\left(i_2^{\mathrm{I}\,0} + i_2^{\mathrm{II}\,0}\right) e^{\alpha_2 f\phi_2^{\mathrm{eq}}}}{\left(i_1^{\mathrm{I}\,0} + i_1^{\mathrm{II}\,0}\right) e^{-(1-\alpha_1)f\phi_1^{\mathrm{eq}}}} \tag{S1-15}$$

The current at mixed potential is:

$$\begin{aligned} i^{\mathrm{mix}} = \left|i_1^{\mathrm{I}} + i_1^{\mathrm{II}}\right| &= \left(i_1^{\mathrm{I}\,0} + i_1^{\mathrm{II}\,0}\right) e^{(1-\alpha_1)f\eta_1^{\mathrm{mix}}} \\ &= \left(i_1^{\mathrm{I}\,0} + i_1^{\mathrm{II}\,0}\right)\left(\frac{\left(i_2^{\mathrm{I}\,0} + i_2^{\mathrm{II}\,0}\right)}{\left(i_1^{\mathrm{I}\,0} + i_1^{\mathrm{II}\,0}\right)} e^{\alpha_2 f\left(\phi_2^{\mathrm{eq}} - \phi_1^{\mathrm{eq}}\right)}\right)^{\frac{1-\alpha_1}{(1-\alpha_1+\alpha_2)}} \end{aligned} \tag{S1-16}$$

Further, we show $\left|\eta_1^{\mathrm{mix}}\right|$ and $\left|\eta_2^{\mathrm{mix}}\right|$ by applying the ϕ^{mix} of Eq. (S1-15):

$$\left|\eta_1^{\mathrm{mix}}\right| = \phi^{\mathrm{mix}} - \phi_1^{\mathrm{eq}} = \frac{\alpha_2}{(1-\alpha_1+\alpha_2)}\left(\phi_2^{\mathrm{eq}} - \phi_1^{\mathrm{eq}}\right) + \frac{1}{(1-\alpha_1+\alpha_2)f} \ln \frac{\left(i_2^{\mathrm{I}\,0} + i_2^{\mathrm{II}\,0}\right)}{\left(i_1^{\mathrm{I}\,0} + i_1^{\mathrm{II}\,0}\right)} \tag{S1-17}$$

$$\left|\eta_2^{\mathrm{mix}}\right| = \phi_2^{\mathrm{eq}} - \phi^{\mathrm{mix}} = \frac{(1-\alpha_1)}{(1-\alpha_1+\alpha_2)}\left(\phi_2^{\mathrm{eq}} - \phi_1^{\mathrm{eq}}\right) - \frac{1}{(1-\alpha_1+\alpha_2)f} \ln \frac{\left(i_2^{\mathrm{I}\,0} + i_2^{\mathrm{II}\,0}\right)}{\left(i_1^{\mathrm{I}\,0} + i_1^{\mathrm{II}\,0}\right)} \tag{S1-18}$$

which shows that the partitioning of total driving force $\phi_2^{\mathrm{eq}} - \phi_1^{\mathrm{eq}}$ mainly depends on the ratio of $\left(i_2^{\mathrm{I}\,0} + i_2^{\mathrm{II}\,0}\right) : \left(i_1^{\mathrm{I}\,0} + i_1^{\mathrm{II}\,0}\right)$ with the logarithm dependence. Another important point is that the transfer coefficient α_1 and α_2 will also contribute to the partitioning.

(c) Linear approximation of Taylor expansion

At sufficient low overpotentials, current can be expressed by a linear expression:

$$i = i^0 f\eta \tag{S1-19}$$

when only zeroth-order and first-order terms of $\exp(x) = 1 + x + \frac{x^2}{2} + \cdots$, are employed, and second-order and higher terms are ignored. The condition of zero net current for forming mixed potential under linear approximation becomes:

$$i_1^{\mathrm{I}\,0} f\eta_1^{\mathrm{mix}} + i_1^{\mathrm{II}\,0} f\eta_1^{\mathrm{mix}} + i_2^{I\,0} f\eta_2^{\mathrm{mix}} + i_2^{II\,0} f\eta_2^{\mathrm{mix}} = 0 \tag{S1-20}$$

Substituting the overpotentials of Eqs. (S1-2) and (S1-3) into Eq. (S1-20) and rearranging it yield:

$$\begin{aligned} &\left(i_1^{\mathrm{I}\,0} + i_1^{\mathrm{II}\,0} + i_2^{\mathrm{I}\,0} + i_2^{\mathrm{II}\,0}\right) f\phi^{\mathrm{mix}} \\ &\qquad = \left(i_1^{\mathrm{I}\,0} + i_1^{\mathrm{II}\,0}\right) f\phi_1^{\mathrm{eq}} + \left(i_2^{\mathrm{I}\,0} + i_2^{\mathrm{II}\,0}\right) f\phi_2^{\mathrm{eq}} \end{aligned} \tag{S1-21}$$

Thus, mixed potential ϕ^{mix} and current at mixed potential can be obtained as follows:

$$\phi^{\mathrm{mix}} = \frac{\left(i_1^{\mathrm{I}\,0} + i_1^{\mathrm{II}\,0}\right)\phi_1^{\mathrm{eq}} + \left(i_2^{\mathrm{I}\,0} + i_2^{\mathrm{II}\,0}\right)\phi_2^{\mathrm{eq}}}{i_1^{\mathrm{I}\,0} + i_1^{\mathrm{II}\,0} + i_2^{\mathrm{I}\,0} + i_2^{\mathrm{II}\,0}} \tag{S1-22}$$

$$i^{\mathrm{mix}} = \left(i_1^{\mathrm{I}\,0} + i_1^{\mathrm{II}\,0}\right) f\eta_1^{\mathrm{mix}} = \left(i_1^{\mathrm{I}\,0} + i_1^{\mathrm{II}\,0}\right)\frac{\left(i_2^{\mathrm{I}\,0} + i_2^{\mathrm{II}\,0}\right)\left(\phi_2^{\mathrm{eq}} - \phi_1^{\mathrm{eq}}\right)}{i_1^{\mathrm{I}\,0} + i_1^{\mathrm{II}\,0} + i_2^{\mathrm{I}\,0} + i_2^{\mathrm{II}\,0}} \tag{S1-23}$$

The ϕ^{mix} of Eq. (S1-22) with the linear approximation greatly simplifies the mathematics for the expression of the absolute value overpotential $\left|\eta_1^{\mathrm{mix}}\right|$ and $\left|\eta_2^{\mathrm{mix}}\right|$:

$$\left|\eta_1^{\mathrm{mix}}\right| = \phi^{\mathrm{mix}} - \phi_1^{\mathrm{eq}} = \frac{\left(i_2^{\mathrm{I}\,0} + i_2^{\mathrm{II}\,0}\right)}{i_1^{\mathrm{I}\,0} + i_1^{\mathrm{II}\,0} + i_2^{\mathrm{I}\,0} + i_2^{\mathrm{II}\,0}}\left(\phi_2^{\mathrm{eq}} - \phi_1^{\mathrm{eq}}\right) \tag{S1-24}$$

$$\left|\eta_2^{\mathrm{mix}}\right| = \phi_2^{\mathrm{eq}} - \phi^{\mathrm{mix}} = \frac{\left(i_1^{\mathrm{I}\,0} + i_1^{\mathrm{II}\,0}\right)}{i_1^{\mathrm{I}\,0} + i_1^{\mathrm{II}\,0} + i_2^{\mathrm{I}\,0} + i_2^{\mathrm{II}\,0}}\left(\phi_2^{\mathrm{eq}} - \phi_1^{\mathrm{eq}}\right) \tag{S1-25}$$

Then, an explicit equation for overpotential partitioning can be obtained as:

$$\left|\eta_1^{\mathrm{mix}}\right| : \left|\eta_2^{\mathrm{mix}}\right| = \frac{1}{i_1^{\mathrm{I}\,0} + i_1^{\mathrm{II}\,0}} : \frac{1}{i_2^{\mathrm{I}\,0} + i_2^{\mathrm{II}\,0}} \tag{S1-26}$$

which suggests that the total driving force $\phi_2^{\mathrm{eq}} - \phi_1^{\mathrm{eq}}$ is divided into $\left|\eta_1^{\mathrm{mix}}\right|$and $\left|\eta_2^{\mathrm{mix}}\right|$in proportion to the reciprocal of exchange currents $\left(i_1^{\mathrm{I}\,0} + i_1^{\mathrm{II}\,0}\right)$ and $\left(i_2^{\mathrm{I}\,0} + i_2^{\mathrm{II}\,0}\right)$, respectively.

Furthermore, the dissipated heat of reaction 1 and 2 can be expressed by:

$$\begin{aligned}
&\eta_1^{\mathrm{mix}}\left(i_1^{\mathrm{I}} + i_1^{\mathrm{II}}\right) : \eta_2^{\mathrm{mix}}\left(i_2^{\mathrm{I}} + i_2^{\mathrm{II}}\right) = \left|\eta_1^{\mathrm{mix}}\right| \times i^{\mathrm{mix}} \;:\; \left|\eta_2^{\mathrm{mix}}\right| \times i^{\mathrm{mix}} \\
&= \left|\eta_1^{\mathrm{mix}}\right| \times \left(i_1^{\mathrm{I}\,0} + i_1^{\mathrm{II}\,0}\right) f\eta_1^{\mathrm{mix}} \;:\; \left|\eta_2^{\mathrm{mix}}\right| \times \left(i_2^{\mathrm{I}\,0} + i_2^{\mathrm{II}\,0}\right) f\eta_2^{\mathrm{mix}} \\
&= \left|\eta_1^{\mathrm{mix}}\right| \;:\; \left|\eta_2^{\mathrm{mix}}\right| = \frac{1}{i_1^{\mathrm{I}\,0} + i_1^{\mathrm{II}\,0}} : \frac{1}{i_2^{\mathrm{I}\,0} + i_2^{\mathrm{II}\,0}}
\end{aligned} \tag{S1-27}$$

which indicates that between reaction 1 and reaction 2, the slower reaction consumes more energy and generate more heat, as it requires the larger overpotential to proceed. Substituting the equations of currents with linear approximation gives the heat production of reaction 1 and 2 on each of components I and II as:

$$\begin{aligned}
&\eta_1^{\mathrm{mix}} i_1^{\mathrm{I}} : \eta_1^{\mathrm{mix}} i_1^{\mathrm{II}} : \eta_2^{\mathrm{mix}} i_2^{\mathrm{I}} : \eta_2^{\mathrm{mix}} i_2^{\mathrm{II}} \\
&= \eta_1^{\mathrm{mix}} \times i_1^{\mathrm{I}\,0} f\eta_1^{\mathrm{mix}} \;:\; \eta_1^{\mathrm{mix}} \times i_1^{\mathrm{II}\,0} f\eta_1^{\mathrm{mix}} \;:\; \eta_2^{\mathrm{mix}} \times i_2^{\mathrm{I}\,0} f\eta_2^{\mathrm{mix}} \;:\; \eta_2^{\mathrm{mix}} \times i_2^{\mathrm{II}\,0} f\eta_2^{\mathrm{mix}} \\
&= \left(\eta_1^{\mathrm{mix}}\right)^2 \times i_1^{\mathrm{I}\,0} \;:\; \left(\eta_1^{\mathrm{mix}}\right)^2 \times i_1^{\mathrm{II}\,0} \;:\; \left(\eta_2^{\mathrm{mix}}\right)^2 \times i_2^{\mathrm{I}\,0} \;:\; \left(\eta_2^{\mathrm{mix}}\right)^2 \times i_2^{\mathrm{II}\,0} \\
&= \left(\frac{1}{i_2^{\mathrm{I}\,0} + i_2^{\mathrm{II}\,0}}\right)^2 \times i_1^{\mathrm{I}\,0} : \left(\frac{1}{i_2^{\mathrm{I}\,0} + i_2^{\mathrm{II}\,0}}\right)^2 \times i_1^{\mathrm{II}\,0} : \left(\frac{1}{i_1^{\mathrm{I}\,0} + i_1^{\mathrm{II}\,0}}\right)^2 \times i_2^{\mathrm{I}\,0} : \left(\frac{1}{i_1^{\mathrm{I}\,0} + i_1^{\mathrm{II}\,0}}\right)^2 \times i_2^{\mathrm{II}\,0} \\
&= \frac{i_1^{\mathrm{I}\,0}}{\left(i_1^{\mathrm{I}\,0} + i_1^{\mathrm{II}\,0}\right)^2} : \frac{i_1^{\mathrm{II}\,0}}{\left(i_1^{\mathrm{I}\,0} + i_1^{\mathrm{II}\,0}\right)^2} : \frac{i_2^{\mathrm{I}\,0}}{\left(i_2^{\mathrm{I}\,0} + i_2^{\mathrm{II}\,0}\right)^2} : \frac{i_2^{\mathrm{II}\,0}}{\left(i_2^{\mathrm{I}\,0} + i_2^{\mathrm{II}\,0}\right)^2}
\end{aligned} \tag{S1-28}$$

However, Eq. (S1-28) indicates that the faster reactions (with higher exchange current) between the same half-reaction on difference components (for example, reaction 1 on component I, i_1^{I}, and reaction 1 on component II, i_1^{II}) will consume more energy and generate more heat because i_1^{I} and i_1^{II} have the same overpotential.

Supplementary Note 2: Adjustment of the overpotential partitioning based on the ratio of the exchange currents

Now, we understand that the total driving force (overpotential) is partitioned between the two half-reactions, primarily based on the exchange current, i^0. To facilitate some

kinetically challenging half-reactions and enhance the net reaction rate by adjusting the overpotential partitioning, it's crucial to comprehend the nature of the exchange current. The exchange current (i^0) is a parameter where the forward and reverse currents are equal and opposite at any given composition. Furthermore, i^0 can be characterized by the activation energy when the reaction is at an equilibrium potential relative to the solution chemistry (*1*). Thus, i^0 is a kinetic parameter and correponds to catalytic activity. Exchange currents ${i_1^{\mathrm{I}}}^0$, ${i_1^{\mathrm{II}}}^0$, ${i_2^{\mathrm{I}}}^0$ and ${i_2^{\mathrm{II}}}^0$ of reaction 1 and 2 on components I and II are expressed using the surface area, substance concentration, frequency factor, and activation energy as:

$${i_1^{\mathrm{I}}}^0 \equiv nA_1^{\mathrm{I}}F\nu_{1\mathrm{f}}^{\mathrm{I}}e^{-\frac{E_1^{\mathrm{I}}}{RT}}{C_{\mathrm{O}_1}}^{(1-\alpha_1)}{C_{\mathrm{R}_1}}^{\alpha_1} \tag{S2-1a}$$

$${i_1^{\mathrm{II}}}^0 \equiv nA_1^{\mathrm{II}}F\nu_{1f}^{\mathrm{II}}e^{-\frac{E_1^{\mathrm{II}}}{RT}}{C_{\mathrm{O}_1}}^{(1-\alpha_1)}{C_{\mathrm{R}_1}}^{\alpha_1} \tag{S2-1b}$$

$${i_2^{\mathrm{I}}}^0 \equiv nA_2^{\mathrm{I}}F\nu_{2f}^{\mathrm{I}}e^{-\frac{E_2^{\mathrm{I}}}{RT}}{C_{\mathrm{O}_2}}^{(1-\alpha_2)}{C_{\mathrm{R}_2}}^{\alpha_2} \tag{S2-1c}$$

$${i_2^{\mathrm{II}}}^0 \equiv nA_2^{\mathrm{II}}F\nu_{2\mathrm{f}}^{\mathrm{II}}e^{-\frac{E_2^{\mathrm{II}}}{RT}}{C_{\mathrm{O}_2}}^{(1-\alpha_2)}{C_{\mathrm{R}_2}}^{\alpha_2} \tag{S2-1d}$$

where A_1^{I}, A_1^{II}, A_2^{I}, and A_2^{II} are the available surface area of the reaction 1 and 2 on components I and II, respectively, C_{O_1}, C_{R_1}, C_{O_2}, and $C_{\mathrm{R}_2}$are the concentration of species O_1, R_1, O_2, and R_2, respectively, $\nu_{1\mathrm{f}}$ and $\nu_{2\mathrm{f}}$ are the frequency factors of the reaction 1 and 2, respectively, E_1^{I}, E_1^{II}, E_2^{I} and E_2^{II} are the activation energies at standard redox potential of the reaction 1 and 2 on components I and II, respectively, and n is the stoichiometric number of electrons consumed in the electrode reaction (in our case, $n = 1$).

There are several ways to tune the overpotential partitioning. However, the fundamental concept remains consistent: all adjustments should aim to modify the ratio of the exchange currents. For simplicity, we assume a single oxidation reaction and a single reduction reaction take place on each catalyst component (while ${i_1^{\mathrm{I}}}^0$ and ${i_2^{\mathrm{II}}}^0$ remain, but ${i_2^{\mathrm{I}}}^0$ and ${i_1^{\mathrm{II}}}^0$ are zero). Then, the two overpotentials can be written as:

$$|\eta_1^{\mathrm{mix}}| = \phi^{\mathrm{mix}} - \phi_1^{\mathrm{eq}} = \frac{1}{f}\ln\frac{e^{\alpha f(\phi_2^{\mathrm{eq}}-\phi_1^{\mathrm{eq}})} + \frac{{i_1^{\mathrm{I}}}^0}{{i_2^{\mathrm{II}}}^0}}{e^{-(1-\alpha)f(\phi_2^{\mathrm{eq}}-\phi_1^{\mathrm{eq}})} + \frac{{i_1^{\mathrm{I}}}^0}{{i_2^{\mathrm{II}}}^0}} \tag{S2-2}$$

$$|\eta_2^{\mathrm{mix}}| = \phi_2^{\mathrm{eq}} - \phi^{\mathrm{mix}} = \frac{1}{f}\ln\frac{e^{(1-\alpha)f(\phi_2^{\mathrm{eq}}-\phi_1^{\mathrm{eq}})} + \frac{{i_2^{\mathrm{II}}}^0}{{i_1^{\mathrm{I}}}^0}}{e^{-\alpha f(\phi_2^{\mathrm{eq}}-\phi_1^{\mathrm{eq}})} + \frac{{i_2^{\mathrm{II}}}^0}{{i_1^{\mathrm{I}}}^0}} \tag{S2-3}$$

In this case, we are particularly interested in the product O_1 generated in the slow oxidation reaction $\mathrm{R}_1 \rightleftarrows \mathrm{O}_1 + e^-$. We can use the polarization curve to understand how to enhance the net reaction rate to get more O_1 by adjusting the overpotential partitioning, as shown in **Supplementary Fig. 1**. Here, the key is to increase the ratio of $(1/{i_1^{\mathrm{I}}}^0)$: $(1/{i_2^{\mathrm{II}}}^0)$. Assumed

a constant value of ${i_1^{\mathrm{I}}}^0$, we can increase the value of ${i_2^{\mathrm{II}}}^0$ of the reduction reaction $\mathrm{O}_2 + e^- \rightleftarrows \mathrm{R}_2$ by modifying the surface area, substance concentration, catalyst component (as specific reactions have varying intrinsic activation energy, frequency factors, and transfer coefficients on different catalysts), as Eq. (S2-1d). As a result, the position of the mixed potential will approach to ϕ_2^{eq}, leading to a different overpotential partitioning based on the ratio of the exchange currents. Essentially, more overpotential is allocated to the slower oxidation reaction, while less overpotential is assigned to the faster reduction reaction. The crucial outcome here is that this adjustment yields a higher net reaction rate or mixed current (i^{mix}).

Supplementary Note 3: Derivation of the Eqs. (23)–(25) for direction of the current flow or electron transfer between components I and II

The flow of currents or electrons into one component is balanced by currents or electrons out of the other component. The direction of current flow or electron transfer between components I and II is determined by whether the current values for each component ($i_1^{\mathrm{I}} + i_2^{\mathrm{I}}$ and $i_1^{\mathrm{II}} + i_2^{\mathrm{II}}$ for compoenet I and II, respectively) are positive or negative. A positive value means current flows out, while a negative value indicates current flows in. For instance, in Case A, the current flows out of component I and into component II, calculated as $i_1^{\mathrm{I}} + i_2^{\mathrm{I}} > 0$ and $i_1^{\mathrm{II}} + i_2^{\mathrm{II}} < 0$. Note here that the current flow between compoenets is what can be measured by using a single cell or H-cell as short-circuited current between anode and cathode, as referenced in (*2–4*). This value differs from the mixed current, $i^{\mathrm{mix}} = \left|i_1^{\mathrm{I}} + i_1^{\mathrm{II}}\right| = \left|i_2^{\mathrm{I}} + i_2^{\mathrm{II}}\right|$.

Noteworthy that both Tafel approximation and linear approximation can be employed to derive the equations for determining the direction of the current flow or electron transfer pathway between component I and II. Remarkably, the results obtained from both approximations are identical.

First, we show the derivation process of Case A that component I is anode and component II is cathode in total (that is, the current flows from component II to I) under Tafel approximation. The currents of reaction 1 and 2 on components I and II under Tafel approximation can be written by using the obtained mixed potential of Eq. (S1-13):

$i_1^{\mathrm{I}} = {i_1^{\mathrm{I}}}^0 \left(\dfrac{\left({i_2^{\mathrm{I}}}^0 + {i_2^{\mathrm{II}}}^0\right)}{\left({i_1^{\mathrm{I}}}^0 + {i_1^{\mathrm{II}}}^0\right) e^{-\alpha_2 f\left(\phi_2^{\mathrm{eq}} - \phi_1^{\mathrm{eq}}\right)}} \right)^{\frac{(1-\alpha_1)}{(1-\alpha_1+\alpha_2)}}$	(S3-1a)
$i_1^{\mathrm{II}} = {i_1^{\mathrm{II}}}^0 \left(\dfrac{\left({i_2^{\mathrm{I}}}^0 + {i_2^{\mathrm{II}}}^0\right)}{\left({i_1^{\mathrm{I}}}^0 + {i_1^{\mathrm{II}}}^0\right) e^{-\alpha_2 f\left(\phi_2^{\mathrm{eq}} - \phi_1^{\mathrm{eq}}\right)}} \right)^{\frac{(1-\alpha_1)}{(1-\alpha_1+\alpha_2)}}$	(S3-1b)
$i_2^{\mathrm{I}} = -{i_2^{\mathrm{I}}}^0 \left(\dfrac{\left({i_2^{\mathrm{I}}}^0 + {i_2^{\mathrm{II}}}^0\right)}{\left({i_1^{\mathrm{I}}}^0 + {i_1^{\mathrm{II}}}^0\right) e^{(1-\alpha_1) f\left(\phi_2^{\mathrm{eq}} - \phi_1^{\mathrm{eq}}\right)}} \right)^{\frac{-\alpha_2}{(1-\alpha_1+\alpha_2)}}$	(S3-1c)

$$i_2^{\mathrm{II}} = -i_2^{\mathrm{II}^0}\left(\frac{\left(i_2^{\mathrm{I}^0}+i_2^{\mathrm{II}^0}\right)}{\left(i_1^{\mathrm{I}^0}+i_1^{\mathrm{II}^0}\right)e^{(1-\alpha_1)f\left(\phi_2^{\mathrm{eq}}-\phi_1^{\mathrm{eq}}\right)}}\right)^{\frac{-\alpha_2}{(1-\alpha_1+\alpha_2)}} \tag{S3-1d}$$

The condition of Case A is that the current flow is from II to I, as expressed by:

$$i_1^{\mathrm{I}} + i_2^{\mathrm{I}} > 0 \text{ and } i_1^{\mathrm{II}} + i_2^{\mathrm{II}} < 0 \tag{S3-2}$$

The former inequation

$$i_1^{\mathrm{I}} + i_2^{\mathrm{I}} > 0 \tag{S3-3}$$

is equivalent to:

$$\frac{i_1^{\mathrm{I}}}{-i_2^{\mathrm{I}}} > 1 \tag{S3-4}$$

Substituting the Eqs. (S3-1a) and (S3-1c) into Eq. (S3-4) gives:

$$\frac{i_1^{\mathrm{I}^0}\left(\frac{\left(i_2^{\mathrm{I}^0}+i_2^{\mathrm{II}^0}\right)}{\left(i_1^{\mathrm{I}^0}+i_1^{\mathrm{II}^0}\right)e^{-\alpha_2 f\left(\phi_2^{\mathrm{eq}}-\phi_1^{\mathrm{eq}}\right)}}\right)^{\frac{(1-\alpha_1)}{(1-\alpha_1+\alpha_2)}}}{i_2^{\mathrm{I}^0}\left(\frac{\left(i_2^{\mathrm{I}^0}+i_2^{\mathrm{II}^0}\right)}{\left(i_1^{\mathrm{I}^0}+i_1^{\mathrm{II}^0}\right)e^{(1-\alpha_1)f\left(\phi_2^{\mathrm{eq}}-\phi_1^{\mathrm{eq}}\right)}}\right)^{\frac{-\alpha_2}{(1-\alpha_1+\alpha_2)}}} > 1 \tag{S3-5}$$

which can be rearranged to:

$$\frac{i_1^{\mathrm{I}^0}\left(\frac{i_2^{\mathrm{I}^0}+i_2^{\mathrm{II}^0}}{i_1^{\mathrm{I}^0}+i_1^{\mathrm{II}^0}}\right)^{\frac{(1-\alpha_1)}{(1-\alpha_1+\alpha_2)}}}{i_2^{\mathrm{I}^0}\left(\frac{i_2^{\mathrm{I}^0}+i_2^{\mathrm{II}^0}}{i_1^{\mathrm{I}^0}+i_1^{\mathrm{II}^0}}\right)^{\frac{-\alpha_2}{(1-\alpha_1+\alpha_2)}}}\frac{e^{\frac{\alpha_2(1-\alpha_1)}{(1-\alpha_1+\alpha_2)}f\left(\phi_2^{\mathrm{eq}}-\phi_1^{\mathrm{eq}}\right)}}{e^{\frac{\alpha_2(1-\alpha_1)}{(1-\alpha_1+\alpha_2)}f\left(\phi_2^{\mathrm{eq}}-\phi_1^{\mathrm{eq}}\right)}} > 1 \tag{S3-6}$$

The exponential term can be cancelled out. Thus, Eq. (S3-6) can be reduced to:

$$\frac{i_1^{\mathrm{I}^0}}{i_2^{\mathrm{I}^0}} > \frac{i_1^{\mathrm{I}^0}+i_1^{\mathrm{II}^0}}{i_2^{\mathrm{I}^0}+i_2^{\mathrm{II}^0}} \tag{S3-7}$$

Similarly, the latter inequation

$$i_1^{\mathrm{II}} + i_2^{\mathrm{II}} < 0 \tag{S3-8}$$

can be equivalent to:

$$\frac{i_1^{\mathrm{II}^0}}{i_2^{\mathrm{II}^0}} < \frac{i_1^{\mathrm{I}^0}+i_1^{\mathrm{II}^0}}{i_2^{\mathrm{I}^0}+i_2^{\mathrm{II}^0}} \tag{S3-9}$$

through Eqs. (S3-1b) and (S3-1d). Then, combining Eqs. (S3-7) and (S3-9) gives:

$$\frac{i_1^{\mathrm{I}^0}}{i_2^{\mathrm{I}^0}} > \frac{i_1^{\mathrm{I}^0}+i_1^{\mathrm{II}^0}}{i_2^{\mathrm{I}^0}+i_2^{\mathrm{II}^0}} > \frac{i_1^{\mathrm{II}^0}}{i_2^{\mathrm{II}^0}} \tag{S3-10}$$

Thus, we can obtain the equation for current direction in Case A:

$$\text{Case A: } \frac{i_1^{\mathrm{I}0}}{i_1^{\mathrm{II}0}} > \frac{i_2^{\mathrm{I}0}}{i_2^{\mathrm{II}0}} \tag{S3-11}$$

The equation for current direction in Case B and C is that the greater-than symbol in Eq. (S3-11) becomes smaller-than sign and equal sign:

$$\text{Case B: } \frac{i_1^{\mathrm{I}0}}{i_1^{\mathrm{II}0}} < \frac{i_2^{\mathrm{I}0}}{i_2^{\mathrm{II}0}} \tag{S3-12}$$

$$\text{Case C: } \frac{i_1^{\mathrm{I}0}}{i_1^{\mathrm{II}0}} = \frac{i_2^{\mathrm{I}0}}{i_2^{\mathrm{II}0}} \tag{S3-13}$$

Next, we derive Case A under linear approximation. The currents under linear approximation can be written by using the obtained mixed potential of Eq. (S1-22):

$$i_1^{\mathrm{I}} = i_1^{\mathrm{I}0} f \frac{\left(i_2^{\mathrm{I}0} + i_2^{\mathrm{II}0}\right)\left(\phi_2^{\mathrm{eq}} - \phi_1^{\mathrm{eq}}\right)}{i_1^{\mathrm{I}0} + i_1^{\mathrm{II}0} + i_2^{\mathrm{I}0} + i_2^{\mathrm{II}0}} \tag{S3-14a}$$

$$i_1^{\mathrm{II}} = i_1^{\mathrm{II}0} f \frac{\left(i_2^{\mathrm{I}0} + i_2^{\mathrm{II}0}\right)\left(\phi_2^{\mathrm{eq}} - \phi_1^{\mathrm{eq}}\right)}{i_1^{\mathrm{I}0} + i_1^{\mathrm{II}0} + i_2^{\mathrm{I}0} + i_2^{\mathrm{II}0}} \tag{S3-14b}$$

$$i_2^{\mathrm{I}} = -i_2^{\mathrm{I}0} f \frac{\left(i_1^{\mathrm{I}0} + i_1^{\mathrm{II}0}\right)\left(\phi_2^{\mathrm{eq}} - \phi_1^{\mathrm{eq}}\right)}{i_1^{\mathrm{I}0} + i_1^{\mathrm{II}0} + i_2^{\mathrm{I}0} + i_2^{\mathrm{II}0}} \tag{S3-14c}$$

$$i_2^{\mathrm{II}} = -i_2^{\mathrm{II}0} f \frac{\left(i_1^{\mathrm{I}0} + i_1^{\mathrm{II}0}\right)\left(\phi_2^{\mathrm{eq}} - \phi_1^{\mathrm{eq}}\right)}{i_1^{\mathrm{I}0} + i_1^{\mathrm{II}0} + i_2^{\mathrm{I}0} + i_2^{\mathrm{II}0}} \tag{S3-14d}$$

The condition

$$i_1^{\mathrm{I}} + i_2^{\mathrm{I}} < 0 \tag{S3-15}$$

can be written as:

$$i_1^{\mathrm{I}0} f \frac{\left(i_2^{\mathrm{I}0} + i_2^{\mathrm{II}0}\right)\left(\phi_2^{\mathrm{eq}} - \phi_1^{\mathrm{eq}}\right)}{i_1^{\mathrm{I}0} + i_1^{\mathrm{II}0} + i_2^{\mathrm{I}0} + i_2^{\mathrm{II}0}} + i_2^{\mathrm{I}0} f \frac{-\left(i_1^{\mathrm{I}0} + i_1^{\mathrm{II}0}\right)\left(\phi_2^{\mathrm{eq}} - \phi_1^{\mathrm{eq}}\right)}{i_1^{\mathrm{I}0} + i_1^{\mathrm{II}0} + i_2^{\mathrm{I}0} + i_2^{\mathrm{II}0}} < 0 \tag{S3-16}$$

Solving Eq. (S3-16) gives:

$$\frac{i_1^{\mathrm{I}0}}{i_2^{\mathrm{I}0}} > \frac{i_1^{\mathrm{I}0} + i_1^{\mathrm{II}0}}{i_2^{\mathrm{I}0} + i_2^{\mathrm{II}0}} \tag{S3-17}$$

which is the same as Eq. (S3-7). Similarly, the condition:

$$i_1^{\mathrm{II}} + i_2^{\mathrm{II}} > 0 \tag{S3-18}$$

can be equivalent to:

$$\frac{i_1^{\mathrm{II}0}}{i_2^{\mathrm{II}0}} < \frac{i_1^{\mathrm{I}0} + i_1^{\mathrm{II}0}}{i_2^{\mathrm{I}0} + i_2^{\mathrm{II}0}} \tag{S3-19}$$

Thus, we can obtain the equation for current direction in Case A by combined Eqs. (S3-17) and (S3-19):

$$\text{Case A: } \frac{i_1^{\mathrm{I}0}}{i_1^{\mathrm{II}0}} > \frac{i_2^{\mathrm{I}0}}{i_2^{\mathrm{II}0}} \tag{S3-20}$$

which is the same as Eq. (S3-11). The similar derivations can be applied to Case B and C. Thus, the same equations for Case A-C to determine the direction of the current flow can be given by both Tafel approximation and linear approximation.

Furthermore, we consider the kinetic nature of Eqs. (S3-11)-(S3-13). Now, introducing the exchange currents of Eqs. (S2-1a)-(S2-1d) into Eq. (S3-11) or (S3-20) gives:

$$\frac{nA_1^{\mathrm{I}}F\nu_{1\mathrm{f}}^{\mathrm{I}}e^{-\frac{E_1^{\mathrm{I}}}{RT}}{C_{\mathrm{O}_1}}^{(1-\alpha_1)}{C_{\mathrm{R}_1}}^{\alpha_1}}{nA_1^{\mathrm{II}}F\nu_{1\mathrm{f}}^{\mathrm{II}}e^{-\frac{E_1^{\mathrm{II}}}{RT}}{C_{\mathrm{O}_1}}^{(1-\alpha_1)}{C_{\mathrm{R}_1}}^{\alpha_1}} > \frac{nA_2^{\mathrm{I}}F\nu_{2\mathrm{f}}^{\mathrm{I}}e^{-\frac{E_2^{\mathrm{I}}}{RT}}{C_{\mathrm{O}_2}}^{(1-\alpha_2)}{C_{\mathrm{R}_2}}^{\alpha_2}}{nA_2^{\mathrm{II}}F\nu_{2\mathrm{f}}^{\mathrm{II}}e^{-\frac{E_2^{\mathrm{II}}}{RT}}{C_{\mathrm{O}_2}}^{(1-\alpha_2)}{C_{\mathrm{R}_2}}^{\alpha_2}} \tag{S3-21}$$

Recognizing that the constant term F and concertration terms in the Eq. (S3-21) can be cancelled, the expression may also be reduced further by assuming that the product of surface area and frequency factor for four currents are the same:

$$A_1^{\mathrm{I}}\nu_{1\mathrm{f}}^{\mathrm{I}} = A_1^{\mathrm{II}}\nu_{1\mathrm{f}}^{\mathrm{II}} = A_2^{\mathrm{I}}\nu_{2\mathrm{f}}^{\mathrm{I}} = A_2^{\mathrm{II}}\nu_{2\mathrm{f}}^{\mathrm{II}} \tag{S3-22}$$

Then, Eq. (S3-21) can be simplified as:

$$\frac{e^{-\frac{E_1^{\mathrm{I}}}{RT}}}{e^{-\frac{E_1^{\mathrm{II}}}{RT}}} > \frac{e^{-\frac{E_2^{\mathrm{I}}}{RT}}}{e^{-\frac{E_2^{\mathrm{II}}}{RT}}} \tag{S3-23}$$

which can be rearranged to:

$$E_1^{\mathrm{I}} - E_2^{\mathrm{I}} < E_1^{\mathrm{II}} - E_2^{\mathrm{II}} \tag{S3-24}$$

Here, the activation energies in Eq. (S3-24) are the activation energy at standard redox potential, which can be defined by the activation energy where no potential difference between electrode and electrolyte and the standard redox potential (*5*). For example, E_1^{I} can be expressed by:

$$E_1^{\mathrm{I}} = {E_1^{\mathrm{I}}}^{\mathrm{f}} - \alpha_1 F\phi_1^0 = {E_1^{\mathrm{I}}}^{\mathrm{r}} + (1-\alpha_1)F\phi_1^0 \tag{S3-25}$$

where ${E_1^{\mathrm{I}}}^{\mathrm{f}}$ and ${E_1^{\mathrm{I}}}^{\mathrm{r}}$ is the forward and reverse activation energy where no potential difference between electrode and electrolyte for reaction 1 on component I, and ϕ_1^0 is its standard redox potential. The same dedinition is applied to E_1^{II}, E_2^{I} and E_2^{II}. Then, Eq. (S3-24) can be rewritten as (here, using forward or reverse activation energy does not matter, and we choose to use forward activation energy term)

$$\left({E_1^{\mathrm{I}}}^{\mathrm{f}} - \alpha_1 F\,\phi_1^0\right) - \left({E_2^{\mathrm{I}}}^{\mathrm{f}} - \alpha_2 F\,\phi_2^0\right) < \left({E_1^{\mathrm{II}}}^{\mathrm{f}} - \alpha_1 F\phi_1^0\right) - \left({E_2^{\mathrm{II}}}^{\mathrm{f}} - \alpha_2 F\phi_2^0\right) \tag{S3-26}$$

which can be reduced into:

$$\text{Case A: } {E_1^{\mathrm{I}}}^{\mathrm{f}} - {E_2^{\mathrm{I}}}^{\mathrm{f}} < {E_1^{\mathrm{II}}}^{\mathrm{f}} - {E_2^{\mathrm{II}}}^{\mathrm{f}} \tag{S3-27}$$

As for case B and C, the derivation process is almost the same, and the equations expressed by forward activation energy are:

$$\text{Case B: } {E_1^{\mathrm{I}}}^{\mathrm{f}} - {E_2^{\mathrm{I}}}^{\mathrm{f}} > {E_1^{\mathrm{II}}}^{\mathrm{f}} - {E_2^{\mathrm{II}}}^{\mathrm{f}} \tag{S3-28}$$

$$\text{Case C: } {E_1^{\mathrm{I}}}^{\mathrm{f}} - {E_2^{\mathrm{I}}}^{\mathrm{f}} = {E_1^{\mathrm{II}}}^{\mathrm{f}} - {E_2^{\mathrm{II}}}^{\mathrm{f}} \tag{S3-29}$$

Eqs. (S3-27)-(S3-29) represent that the direction of the current flow or electron transfer is determined by value of the difference of the intrinsic activation energy.

Supplementary Note 4: Estimation of error when using Tafel and linear approximations

By solving the mixed potential condition that the net current is zero numerically: $i_1^{\mathrm{I}} + i_1^{\mathrm{II}} + i_2^{\mathrm{I}} + i_2^{\mathrm{II}} = 0$, one can get the exact mixed potential value of the system with the known values of exchange current, the equilibrium potentials and transfer coefficients of each reaction. By comparing the direct numerical result of mixed potential (denoted $\phi_{\mathrm{num}}^{\mathrm{mix}}$) with the approximate forms of mixed potential ($\phi_{\mathrm{Tafel}}^{\mathrm{mix}}$ using Tafel approximation and $\phi_{\mathrm{Linear}}^{\mathrm{mix}}$ using linear approximation), we can estimate the error and then determine the suitability of different approximations for varying systems.

Which approximation method is more suitable mainly depends on the exchange current of the two half-reactions for a specific mixed-potential-driven catalytic reaction system. In other words, the ratio of ${i_2^{\mathrm{I}}}^0 + {i_2^{\mathrm{II}}}^0$ and ${i_1^{\mathrm{I}}}^0 + {i_1^{\mathrm{II}}}^0$ is the key point to evalute the suitability of approximations for one system. For simplicity's sake, we can fix the value of ${i_1^{\mathrm{I}}}^0 + {i_1^{\mathrm{II}}}^0$ and change the value of ${i_2^{\mathrm{I}}}^0 + {i_2^{\mathrm{II}}}^0$. The exact values of parameters are shown in **Supplementary Table 1**. The exact numerical results of $\phi_{\mathrm{num}}^{\mathrm{mix}}$, $\phi_{\mathrm{Tafel}}^{\mathrm{mix}}$ and $\phi_{\mathrm{Linear}}^{\mathrm{mix}}$ can be obtained based on the above given parameters, as shown in **Supplementary Fig. 2**.

It is not surprising that linear approximation performs significantly better than Tafel approximation and is nearly consistent with the actual value in the case of **Supplementary Fig. 2A**. The difference between ϕ_1^{eq} and ϕ_2^{eq} is very small and the mixed potential must locate between ϕ_1^{eq} and ϕ_2^{eq}. Consequently, the overpotentials are sufficiently small, satisfying the condition for the application of the linear approximation. Additionally, we exmine a mixed-potential-driven catalytic reaction system characterized by a larger equilibrium potential difference. Therefore, we modify ϕ_1^{eq} from 0.4 V to 0.1 V to check the error when the difference between ϕ_1^{eq} and ϕ_2^{eq} is large. All other parameter values remian unchanged. The comparation of $\phi_{\mathrm{num}}^{\mathrm{mix}}$, $\phi_{\mathrm{Tafel}}^{\mathrm{mix}}$ and $\phi_{\mathrm{Linear}}^{\mathrm{mix}}$ under these conditions is presented in **Supplementary Fig. 2B**. It is clear that Tafel approximation is much better when the difference between ϕ_1^{eq} and ϕ_2^{eq} is large. However, if the ratio of $({i_2^{\mathrm{I}}}^0 + {i_2^{\mathrm{II}}}^0):({i_1^{\mathrm{I}}}^0 + {i_1^{\mathrm{II}}}^0)$ is very large or small, Tafel approximation will give a mixed potential that is outside the acceptable range, which should have located between ϕ_1^{eq} and ϕ_2^{eq}, as shown in both **Supplementary Fig. 2A** and **B**. In this case, we have to employ linear approximation to obtain the mixed potential when the ratio of $({i_2^{\mathrm{I}}}^0 + {i_2^{\mathrm{II}}}^0):({i_1^{\mathrm{I}}}^0 + {i_1^{\mathrm{II}}}^0)$ is very large or small whatever the difference between ϕ_1^{eq} and ϕ_2^{eq}.

Supplementary Note 5: Derivation of Eq. (30) for the energy conversion pathway in the mixed-potential-driven catalysis

The change of the total Gibbs free energy of the reaction system, at constant presure and temperature, for the net reaction Equation (3) in the main text is expressed by:

$$\mathrm{d}G_{\mathrm{sys}} = \mu_{\mathrm{O}_1}\mathrm{d}N_{\mathrm{O}_1} + \mu_{\mathrm{R}_2}\mathrm{d}N_{\mathrm{R}_2} + \mu_{\mathrm{R}_1}\mathrm{d}N_{\mathrm{R}_1} + \mu_{\mathrm{O}_2}\mathrm{d}N_{\mathrm{O}_2} \qquad \text{(S5-1)}$$

where μ_{O_1}, μ_{R_2}, μ_{R_1}, and μ_{O_2} are the chemical potential of the species O_1, R_2, R_1, and O_2, respectively, and $\mathrm{d}N_{O_1}$, $\mathrm{d}N_{R_2}$, $\mathrm{d}N_{R_1}$, and $\mathrm{d}N_{O_2}$ are the change in mole numbers of the species O_1, R_2, R_1, and O_2, respectively. The extent of reaction is defined as:

$$\mathrm{d}\xi \equiv -\mathrm{d}N_{O_1} = -\mathrm{d}N_{R_2} = \mathrm{d}N_{R_1} = \mathrm{d}N_{O_2} \quad \text{(S5-2)}$$

In general, we have:

$$\mathrm{d}\xi \equiv \frac{\mathrm{d}N_{\mathrm{k}}}{\nu_{\mathrm{k}}} \quad \text{(S5-3)}$$

where ν_{k} is a stoichiometric coefficient of species k in the chemical reaction, which is defined to be positive when species k is a "product" in the chemical reaction, negative when species k is a "reactant" of the chemical reaction (*6*). Then, the rate of chemical reaction can be written as:

$$v = \frac{\mathrm{d}\xi}{\mathrm{d}t}\ (\mathrm{mol\ s^{-1}}) \quad \text{(S5-4)}$$

The Gibbs free energy change of the net reaction, ΔG_{r}, is obtained as the derivative the total Gibbs free energy of the reaction system with respect to the extent of reaction as follows:

$$\Delta G_{\mathrm{r}} \equiv \frac{\mathrm{d}G_{\mathrm{sys}}}{\mathrm{d}\xi} = \sum_{\mathrm{k}} \nu_{\mathrm{k}}\mu_{\mathrm{k}} \quad \text{(S5-5)}$$

where μ_{k} is the chemical potential for species k. Equation (S5-5) indicate that ΔG_{r} is the chemical potential difference between reactants and products (*7*, *8*).

De Donder formulated the thermodynamics of irreversible chemical transformations and was able to relate the "uncompensated heat" in a chemical reaction to the affinity A, defined as the driving force for chemical reaction and given by chemical potential difference between reactants and products (*9*). In our manuscript, we define the total driving force is Gibbs free energy drop of the net reaction, which is also the chemical potential difference between reactants and products. Thus, one can write:

$$-\Delta G_{\mathrm{r}} \equiv A \equiv -\left(\frac{\mathrm{d}G_{\mathrm{sys}}}{\mathrm{d}\xi}\right)_{p,T} = -\sum_{\mathrm{k}} \nu_{\mathrm{k}}\mu_{\mathrm{k}} \quad \text{(S5-6)}$$

The driving force $-\Delta G_{\mathrm{r}}$ can be illustrated as the slope of $G\ vs.\ \xi$ plot at constant pressure and temperature, as shown in **Fig. S3**. Since chemical potentials vary with the composition of a reacting mixture, the slope of the $G\ vs.\ \xi$ plot changes as the reaction proceeds. A reaction that spontaneously converts reactants to products has a negative slope, which means that the driving force is above zero ($-\Delta G_{\mathrm{r}} > 0$). Also noteworthy is that $-\Delta G_{\mathrm{r}}$ is a concept that relates irreversible chemical reactions to entropy production, and $-\Delta G_{\mathrm{r}}$ drives the reactions irreversibly towards equilibrium. At equilibrium, the driving force $-\Delta G_{\mathrm{r}} = 0$ as the Gibbs free energy of the system reaches to its minimum value G_{min} at ξ_{eq} (*8*).

The Gibbs free energy drop of the net reaction can be converted into the corresponding equilibrium potential difference, which is the electromotive force of the reaction considered (*10*, *11*). The relationship of the Gibbs free energy drop, the equilibrium potential difference, and overpotentials can be expressed by:

$$-\Delta G_{\mathrm{r}} = nF\left(\phi_2^{\mathrm{eq}} - \phi_1^{\mathrm{eq}}\right) = n'F\left(\phi_2^{\mathrm{eq}} - \phi^{\mathrm{mix}} + \phi^{\mathrm{mix}} - \phi_1^{ocp}\right) = n'F\left(\left|\eta_1^{\mathrm{mix}}\right| + \left|\eta_2^{\mathrm{mix}}\right|\right) \quad \text{(S5-7)}$$

where n' is the electron transfer number in the rate limiting step, respectively. We assumed $n = n' = 1$ in the main text for simplicity so that we get Eq. (21). The following equation demonstrates the proportionality between the rate of the chemical reaction and the reaction current:

$$v = \frac{\mathrm{d}\xi}{\mathrm{d}t} = \frac{i}{nF} \quad \text{(S5-8)}$$

where $n = 1$ in our case. Thus, one can write the equation for the conversion from Gibbs free energy to electric energy in the mixed-potential-driven catalysis as:

$$-\frac{\mathrm{d}G_{\mathrm{sys}}}{\mathrm{d}t} = (-\Delta G_{\mathrm{r}})\frac{\mathrm{d}\xi}{\mathrm{d}t} = \frac{-\Delta G_{\mathrm{r}}}{F}F\frac{\mathrm{d}\xi}{\mathrm{d}t} = (\phi_2^{\mathrm{eq}} - \phi_1^{\mathrm{eq}})F\frac{\mathrm{d}\xi}{\mathrm{d}t} = (|\eta_1^{\mathrm{mix}}| + |\eta_2^{\mathrm{mix}}|)i^{\mathrm{mix}} \quad \text{(S5-9)}$$

Supplementary Note 6: Expansion for general system with external electrochemical work

The framework of mixed-potential-driven catalysis can be extended to systems that perform electrochemical work for external parties. A typical example is a fuel cell in operation. In biological systems, enzyme complexes such as complexes I and IV of the mitochondrial respiratory chain are classified as this system (*12*). In complexes, anodic and cathodic reactions are paired, and part of the difference in their equilibrium potential is used for the electrochemical work of pumping protons out of the matrix and into the inner membrane space (*13*). Letting the electrochemical work extracted externally be $\Delta\phi^{\mathrm{work}}$, the total overpotential that can be used to drive the reactions is given by $\phi_2^{\mathrm{eq}} - \phi_1^{\mathrm{eq}} - \Delta\phi^{\mathrm{work}}$, as shown in **Supplementary Fig. 4**. The overpotential distributed to anodic reaction 1 and cathodic reaction 2 is given by solving $i_1^{\mathrm{I}} + i_1^{\mathrm{II}} + i_2^{\mathrm{I}} + i_2^{\mathrm{II}} = 0$ under linear approximation as follows:

$$|\eta_1^{\mathrm{mix}}| : |\eta_2^{\mathrm{mix}}| = \frac{\left(i_2^{\mathrm{I}0} + i_2^{\mathrm{II}0}\right)}{i_1^{\mathrm{I}0} + i_1^{\mathrm{II}0} + i_2^{\mathrm{I}0} + i_2^{\mathrm{II}0}}(\phi_2^{\mathrm{eq}} - \phi_1^{\mathrm{eq}} - \Delta\phi^{\mathrm{work}}) : \frac{\left(i_1^{\mathrm{I}0} + i_1^{\mathrm{II}0}\right)}{i_1^{\mathrm{I}0} + i_1^{\mathrm{II}0} + i_2^{\mathrm{I}0} + i_2^{\mathrm{II}0}}(\phi_2^{\mathrm{eq}} - \phi_1^{\mathrm{eq}} - \Delta\phi^{\mathrm{work}})$$
$$= \frac{1}{i_1^{\mathrm{I}0} + i_1^{\mathrm{II}0}} : \frac{1}{i_2^{\mathrm{I}0} + i_2^{\mathrm{II}0}} \quad \text{(S6-1)}$$

As can be seen from equation (S6-1), the principle of overpotential partitioning remains the same even when the reaction system performs external electrochemical work. The sum of the overpotentials $\phi_2^{\mathrm{eq}} - \phi_1^{\mathrm{eq}} - \Delta\phi^{\mathrm{work}}$ is distributed between reaction 1 and reaction 2 according to the ratio of the exchange currents.

Supplementary Note 7: Effects of mass transport

In the case of no mass-transfer effects, the surface concentrations do not differ appreciably from the bulk values and the current increases rapidly with greater overpotential due to dominating exponential factors. However, with the consideration of the mass transport effect, one must distinguish carefully between bulk and surface concentrations. In the net reaction of Eq. (3), the reactants are $\mathrm{R_1}$ and $\mathrm{O_2}$, so that their surface concentrations are smaller than their bulk concentrations. In contrast, the surface concentrations of the products, $\mathrm{O_1}$ and $\mathrm{R_2}$, are greater than their bulk concentrations.

We consider an extreme case where one reaction is solely charge-transfer controlled and one reaction is completely diffusion limited. The mass transfer effects can be neglected in the anodic half-reaction 1. However, the reaction current of the cathodic half-reaction 2 is expressed as a diffusion limiting current. With consideration of mass transport, the current against potential curve levels off at extreme overpotentials. In these level regions, the current is limited by mass transport rather than activation kinetics. Indeed, this situation is applicable in the cases when (i) the difference between equilibrium potentials is large (i.e., the driving force, $\phi_2^{\mathrm{eq}} - \phi_1^{\mathrm{eq}}$, is large), (ii) the value of exchange currents is large (i.e., polarization curves rise steeply), and (iii) the concentrations of the reactants are low.

We can use illustrated polarization curves to understand how the mixed potential and reaction current will change, as shown in **Fig. S5**. Then, the reaction current at mixed potential i^{mix} is equal to the diffusion limiting current of half-reaction 2. As a result, the mixed potential would move away from reaction 2 and toward reaction 1 compared to the case where there is no diffusion effect in reaction 2. The mixed potential is determined by the equilibrium potential and exchange current of half-reaction 1 and the mass transfer coefficients and reactant concentration of half-reaction 2.

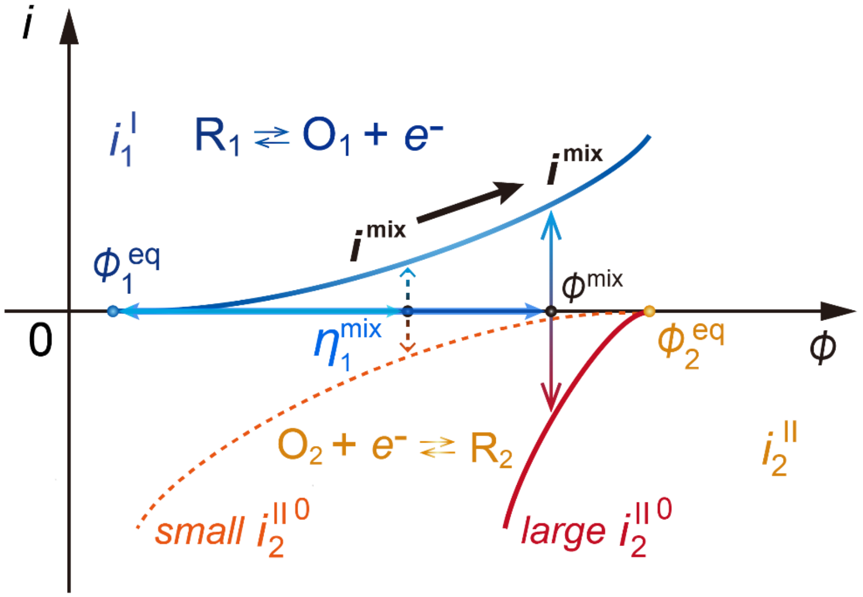

Supplementary Figure 1. Illustration of the polarization curves to understand how to enhance the net reaction rate by adjusting the overpotential partitioning. The polarization curves for half-reaction 1, $\mathbf{R_1} \rightleftarrows \mathbf{O_1} + \boldsymbol{e}^-$, on component I ($\boldsymbol{i_1^{\mathrm{I}}}$) and half-reaction 2, $\mathbf{O_2} + \boldsymbol{e}^- \rightleftarrows \mathbf{R_2}$, on component II ($\boldsymbol{i_2^{\mathrm{II}}}$). The driving force of knetically difficult half-reaction 1, $\boldsymbol{\eta_1^{\mathrm{mix}}}$, can significantly be increased by coupling the more kinetically favorable half-reaction 2 which has large $\boldsymbol{i_2^{\mathrm{II}\,0}}$. In other words, more overpotential is given to the kinetically difficult half-reaction 1 and thus enhances the net reaction rate according to the ratio of $\boldsymbol{i_1^{\mathrm{I}\,0}} : \boldsymbol{i_2^{\mathrm{II}\,0}}$.

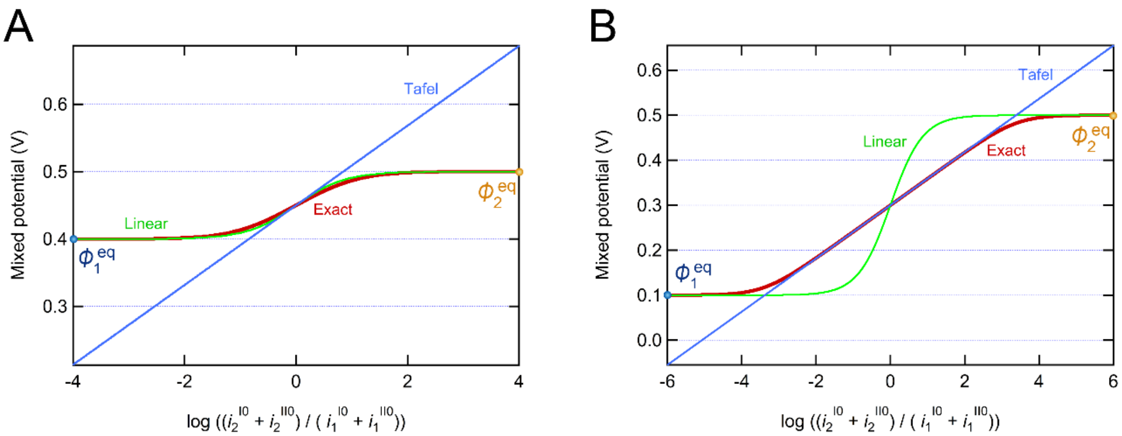

Supplementary Figure 2. The value $\boldsymbol{\phi_{\mathrm{num}}^{\mathrm{mix}}}$, $\boldsymbol{\phi_{\mathrm{Tafel}}^{\mathrm{mix}}}$ and $\boldsymbol{\phi_{\mathrm{Linear}}^{\mathrm{mix}}}$ as the change of ratio of $(\boldsymbol{i_2^{\mathrm{I}}}^{\mathbf{0}} + \boldsymbol{i_2^{\mathrm{II}}}^{\mathbf{0}}):(\boldsymbol{i_1^{\mathrm{I}}}^{\mathbf{0}} + \boldsymbol{i_1^{\mathrm{II}}}^{\mathbf{0}})$ when the difference between $\boldsymbol{\phi_1^{\mathrm{ocp}}}$ and $\boldsymbol{\phi_2^{\mathrm{ocp}}}$ is (A) 0.1 V ($\boldsymbol{\phi_1^{\mathrm{ocp}} = 0.4\ \mathrm{V}}$ and $\boldsymbol{\phi_2^{\mathrm{ocp}} = 0.5\ \mathrm{V}}$) and (B) 0.4 V ($\boldsymbol{\phi_1^{\mathrm{ocp}} = 0.1\ \mathrm{V}}$ and $\boldsymbol{\phi_2^{\mathrm{ocp}} = 0.5\ \mathrm{V}}$).

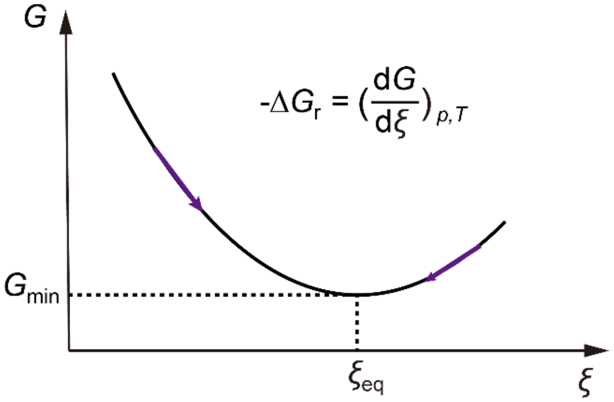

Supplementary Figure 3. As the reaction proceeds (represented by motion from left to right along the horizontal axis: Extent of reaction $\boldsymbol{\xi}$), the slope of the Gibbs free energy changes. Equilibrium corresponds to the zero slope where the extent of reaction goes to $\boldsymbol{\xi_{eq}}$ and Gibbs free energy of the system at its minimum value $\boldsymbol{G_{min}}$.

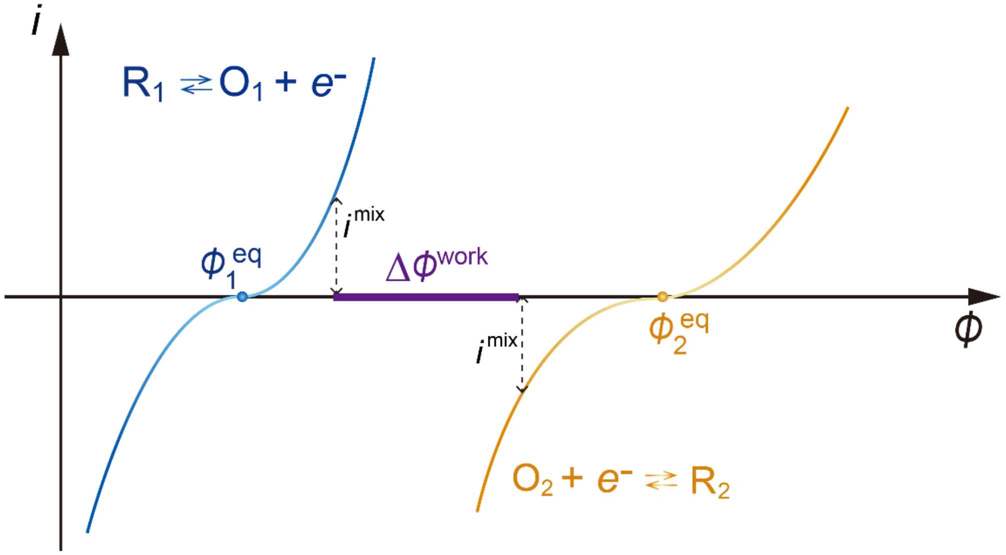

Supplementary Figure 4. Schematic diagram of the polarization curves and reaction current when there is external electrochemical work $\Delta\boldsymbol{\phi}^{\mathbf{work}}$ in the mixed-potential-driven catalysis.

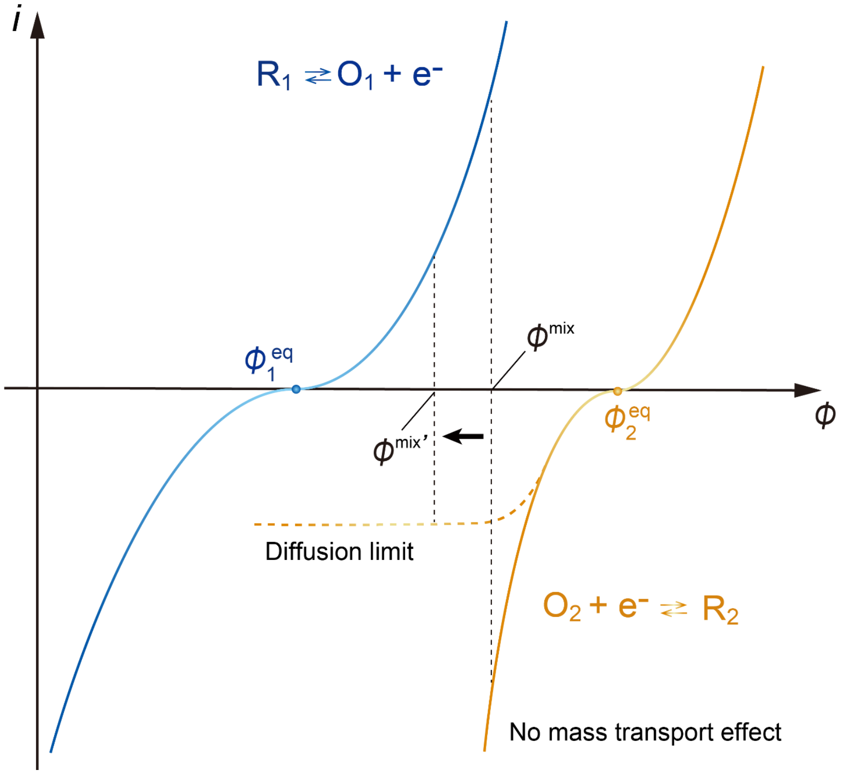

Supplementary Figure 5. Schematic plots of polarization curves for two half-reactions illustrate the mass transfer effect. Half-reaction 1 is solely charge-transfer controlled. The solid and dash lines of half-reaction 2 represent the currents of no mass transport effect and diffusion limiting, respectively. In this case, the mixed potential would shift to lower potentials from $\boldsymbol{\phi}^{\mathbf{mix}}$ to $\boldsymbol{\phi}^{\mathbf{mix}'}$.

Supplementary Table 1. The set values of parameters for solving the numerical value of mixed potential

**Parameters**	**Values**
${i_1^{\mathrm{I}}}^0 + {i_1^{\mathrm{II}}}^0$	10^{-3} A cm^{-2}
${i_2^{\mathrm{I}}}^0 + {i_2^{\mathrm{II}}}^0$	$10^{-9} \sim 10^{3}$ A cm^{-2}
ϕ_1^{eq}	0.4 V (Fig. S2A); 0.1 V (Fig. S2B);
ϕ_2^{eq}	0.5 V
α_1	0.5
α_2	0.5

Supplementary References:

1. A. J. Bard, L. R. Faulkner, *Electrochemical Methods: Fundamentals and Applications* (John Wiley & Sons, ed. 2, 2001).
2. X. Huang, O. Akdim, M. Douthwaite, K. Wang, L. Zhao, R. J. Lewis, S. Pattisson, I. T. Daniel, P. J. Miedziak, G. Shaw, D. J. Morgan, S. M. Althahban, T. E. Davies, Q. He, F. Wang, J. Fu, D. Bethell, S. McIntosh, C. J. Kiely, G. J. Hutchings, Au–Pd separation enhances bimetallic catalysis of alcohol oxidation. *Nature* **603**, 271–275 (2022).
3. H. An, G. Sun, M. J. Hülsey, P. Sautet, N. Yan, Demonstrating the Electron-Proton-Transfer Mechanism of Aqueous Phase 4-Nitrophenol Hydrogenation Using Unbiased Electrochemical Cells. *ACS Catal* **12**, 15021–15027 (2022).
4. K. Takeyasu, Y. Katane, N. Miyamoto, M. Yan, J. Nakamura, Experimental Verification of Mixed-potential-driven Catalysis. *e-Journal of Surface Science and Nanotechnology* **21**, 164–168 (2022).
5. J. O. M. Bockris, S. U. M. Khan, *Surface Electrochemistry: A Molecular Level Approach* (Springer, 1993).
6. M. Boudart, G. Djega-Mariadassou, *Kinetics of Heterogeneous Catalytic Reactions* (Princeton University Press, New Jersey, 1984).
7. I. Prigogine, *Introduction to Thermodynamics of Irreversible Processes* (John Wiley & Sons, ed. 3, 1968).
8. D. Kondepudi, I. Prigogine, *Modern Thermodynamics: From Heat Engines to Dissipative Structures* (John Wiley & Sons, 1998).
9. T. de Donder, P. van Rysselberghe, *Thermodynamic Theory of Affinity: A Book of Principles* (Stanford University Press; Milford, Oxford university press, 1936).
10. J. O. Bockris, Amulya K. N. Reddy, *Modern Electrochemistry 2B: Electrodics in Chemistry, Engineering, Biology and Environmental Science* (Kluwer Academic Publishers, ed. 2, 2001).
11. L. Lazzari, “General aspects of corrosion” in *Encyclopaedia of Hydrocarbons* (Eni: Istituto della Enciclopedia italiana, 2005), pp. 485–505.
12. L. A. Smith, M. W. Glasscott, K. J. Vannoy, J. E. Dick, Enzyme Kinetics via Open Circuit Potentiometry. *Anal Chem* **92**, 2266–2273 (2020).
13. A. M. Bertholet, Y. Kirichok, Mitochondrial H Leak and Thermogenesis. *Annu Rev Physiol* **84**, 381–407 (2022).
